# Genomes as geography: using GIS technology to build interactive genome feature maps

**DOI:** 10.1186/1471-2105-7-416

**Published:** 2006-09-19

**Authors:** Mary E Dolan, Constance C Holden, M Kate Beard, Carol J Bult

**Affiliations:** 1 National Center for Geographic Information and Analysis, University of Maine, Orono, ME 04469, USA; 2 The Jackson Laboratory, Bar Harbor, ME 04609, USA

## Abstract

**Background:**

Many commonly used genome browsers display sequence annotations and related attributes as horizontal data tracks that can be toggled on and off according to user preferences. Most genome browsers use only simple keyword searches and limit the display of detailed annotations to one chromosomal region of the genome at a time. We have employed concepts, methodologies, and tools that were developed for the display of geographic data to develop a Genome Spatial Information System (GenoSIS) for displaying genomes spatially, and interacting with genome annotations and related attribute data. In contrast to the paradigm of horizontally stacked data tracks used by most genome browsers, GenoSIS uses the concept of registered spatial layers composed of spatial objects for integrated display of diverse data. In addition to basic keyword searches, GenoSIS supports complex queries, including spatial queries, and dynamically generates genome maps. Our adaptation of the geographic information system (GIS) model in a genome context supports spatial representation of genome features at multiple scales with a versatile and expressive query capability beyond that supported by existing genome browsers.

**Results:**

We implemented an interactive genome sequence feature map for the mouse genome in GenoSIS, an application that uses ArcGIS, a commercially available GIS software system. The genome features and their attributes are represented as spatial objects and data layers that can be toggled on and off according to user preferences or displayed selectively in response to user queries. GenoSIS supports the generation of custom genome maps in response to complex queries about genome features based on both their attributes and locations. Our example application of GenoSIS to the mouse genome demonstrates the powerful visualization and query capability of mature GIS technology applied in a novel domain.

**Conclusion:**

Mapping tools developed specifically for geographic data can be exploited to display, explore and interact with genome data. The approach we describe here is organism independent and is equally useful for linear and circular chromosomes. One of the unique capabilities of GenoSIS compared to existing genome browsers is the capacity to generate genome feature maps dynamically in response to complex attribute and spatial queries.

## Background

Biomedical researchers and geographers both face formidable challenges in trying to identify meaningful patterns in the rapidly growing volumes of data and information. Both disciplines rely heavily on the use of maps for abstract representations of data. Maps are particularly useful in these domains because humans are adept at extracting patterns and information from graphical representations of complex data.

Among biologists, web-based genome browsers such as the UCSC Genome Browser [1] and Ensembl [2] are popular community resources for organizing and integrating diverse kinds of biological annotations and attributes that can be mapped to the genome sequence of an organism. Other graphical genome representation tools such as Apollo [3] and Sockeye [4] are popular for specialized applications in the areas of sequence annotation and comparative genomics, respectively. In addition, software such as the Generic Genome Browser, that allows individual investigators to implement their own genome browsers, has been widely used for creating browsable genome maps for diverse organisms [5]. While there are differences in representation and functionality among these genome browsers they all map genome features and their biological attributes to a common genome framework using nucleotide coordinates. The browsers and software tools listed above also share a common visualization mechanism in which different data sets are displayed as horizontal "tracks" that can be toggled on and off according to the interests and preferences of the user. The one exception to this paradigm is NCBI's Map Viewer [6] which supports the simultaneous display of maps built using different underlying coordinate spaces (genetic and genomic maps, for example) and displays maps in a vertical orientation instead of horizontally.

**Figure 1:**
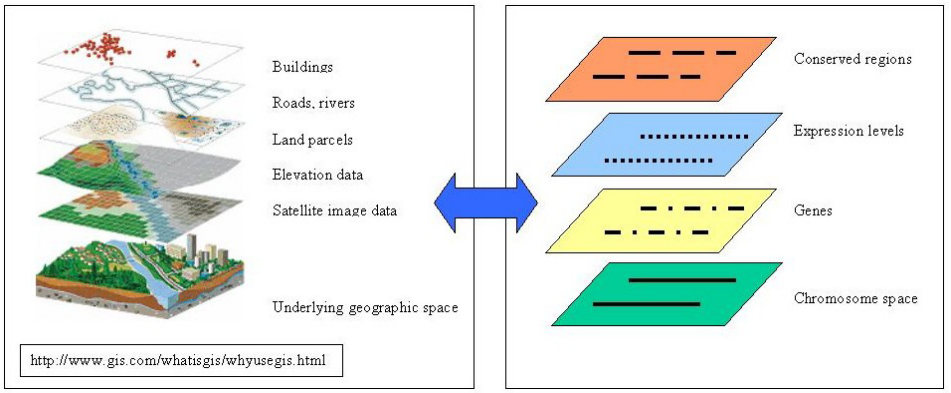
**The GIS paradigm of the map layers can be applied to the integration and visualization of genome data**. For a typical geo-referenced map (left) diverse geographic data (road systems, topography, building positions) can be combined by the relation of each data set to the underlying space defined by geographic location. In a similar way diverse genomic data (right) can be combined by the relationship of each data type to an underlying genome coordinate space or to the features in that space.

In geographic information systems (GIS), maps are created and displayed using 2D (or 3D) coordinate reference systems in a given coordinate space [7]. Different types of geographic features (e.g., cities, rivers, rainfall) are characterized individually and typically stored as different map layers (Figure 1). By employing a common spatial reference system, layers are georegistered and can be overlaid on each other to visually evaluate the spatial distribution of features. Organizing data as spatially referenced layers provides flexibility to select and combine layers in various ways. Through the combination of georegistered layers geographers can evaluate what kinds of feature tend to co-localize or to explain the presence of one feature as a consequence of its spatial relationship to another feature.

In GIS, support for query and display is tightly integrated (i.e. a map is a response to a query). GIS supports spatial selection queries on individual features within a layer with the result that features meeting the query constraints are highlighted on the map. Spatial join queries are a particularly powerful GIS function that allows the user to query on spatial relations among features across map layers.

An example of a typical GIS spatial query is a query for all houses that are priced under $500,000, within School District A and less than 5 miles from a highway. This query executes a proximity query on houses and roads and a spatial containment query on houses and school district A. The result is a map highlighting only those houses satisfying the attribute constraint (price< $500,000) and the two spatial relationship constraints. An example of a spatial join query is a query for all houses with school age children and bus stops within School District A. The query returns the combined set of selected houses and bus stops falling in District A.

The Genome Spatial Information System (GenoSIS) [8] we present here adapts the GIS model to support the spatial representation of genome features. GenoSIS employs GIS functions for panning and zooming, highlighting features of interest or filtering those with certain properties; and employs standard cartographic techniques for encoding variables using graphic symbols (shape, color, etc) [9]. As in other genome browsers we define a genome map space by nucleotide coordinates along chromosomes. Unlike their treatment in other browsers, in GenoSIS, genome features are all defined as spatial objects. In this "spatial genome" representation users have the flexibility to interact with genome features as layers, as individual features within a layer, or as collections of features across layers (Figure 1).

GenoSIS allows users to build interactive genome maps using queries that integrate information about the biological attributes and spatial relationships among genome features. The results of queries in GenoSIS are themselves maps that can be saved and further refined. The functionality for data display, exploration and interaction inherent in GenoSIS is unique among existing genome browsers making it a powerful tool for data mining.

## Implementation

We have implemented GenoSIS using ArcGIS [10], a spatial information system commonly used for geo-referenced data. ArcGIS is a commercial software system that is available in desktop and server configurations. Map files that are published from ArcGIS can be read on Windows platforms by a freely available software tool, ArcReader [11] that supports map browsing but does not support the dynamic generation of maps in response to complex queries. ArcReader is also available from ESRI (Environmental Systems Research Institute [12]) for Linux and Solaris platforms for a nominal fee.

The chromosome forms the foundation layer of our implementation. Each chromosome within the layer is represented as a linear spatial object with a unique identifier and a length (in bp). The arrangement (placement and separation) of the chromosome line objects creates a 2D space. The coordinate space defined by the chromosome arrangement provides the spatial reference system and all other genome features are "georegistered" to this space.

To apply GenoSIS to analysis of the mouse genome we used genome features and attributes obtained from the following sources:

1. ***genes***: mouse genes, their chromosome position, and the start and end coordinates (NCBI Build 34 of the mouse genome) along the genome were obtained from the Mouse Genome Informatics (MGI) database [13] public ftp site [14]

2. ***gene_structure***: coordinate data (NCBI Build 34) for defining gene structure (i.e., intron-exon boundaries) for mouse genes was downloaded from NCBI [15]

3. ***GO_function***: the annotation of mouse genes to high level terms in the Gene Ontology [16] were generated specifically for this study and are available online [17]

4. ***human_orthologs***: annotations about which mouse genes have human orthologs were downloaded from the MGI ftp site [18]

5. ***gene_expression***: a data set of developmental stage and tissue-specific expression levels for mouse genes [19] was downloaded from NCBI's Gene Expression Omnibus [20]: GDS592 [21]

6. ***TFBS***: transcription factor binding sites used for this manuscript are for the RBP-J protein and were generated by one of the authors (CJB) using a string matching algorithm of the canonical transcription factor binding site for Build 34 of the mouse genome sequence.

The *genes*, *gene_structure*, and *TFBS *were created as spatial objects georegistered to the chromosome space by genome coordinates, and they can be displayed directly as layers to the chromosome base. The *GO_function*, *human_orthologs*, and *gene_expression *are treated as attributes associated with individual genes or sets of genes. Each of these files was linked to the gene table by joins on the MGI gene identifier. For example, the *GO_function *data set is a two-column table containing MGI gene identifiers and GO functional annotations; this table was joined (within ArcGIS) to the *genes *data set based on shared MGI gene identifiers.

## Results

**Figure 2:**
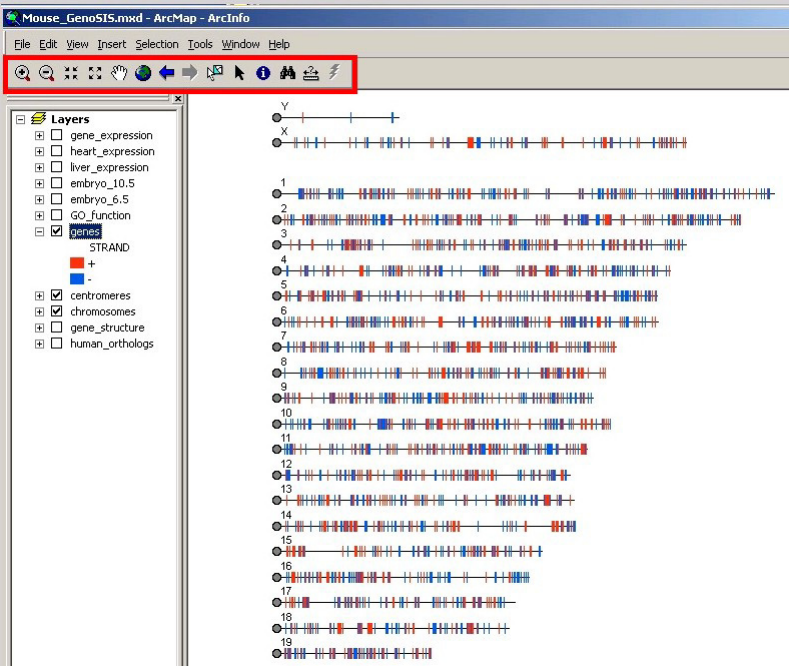
**The mouse genome displayed in GenoSIS**. In this figure, the chromosome, centromere, and gene layers have been activated (see left panel). The ArcGIS toolbar (area boxed in red) shows some of the many built-in tools of the underlying GIS software: zoom (magnifying glass), pan (hand), identify, search, and measure.

**Figure 3:**
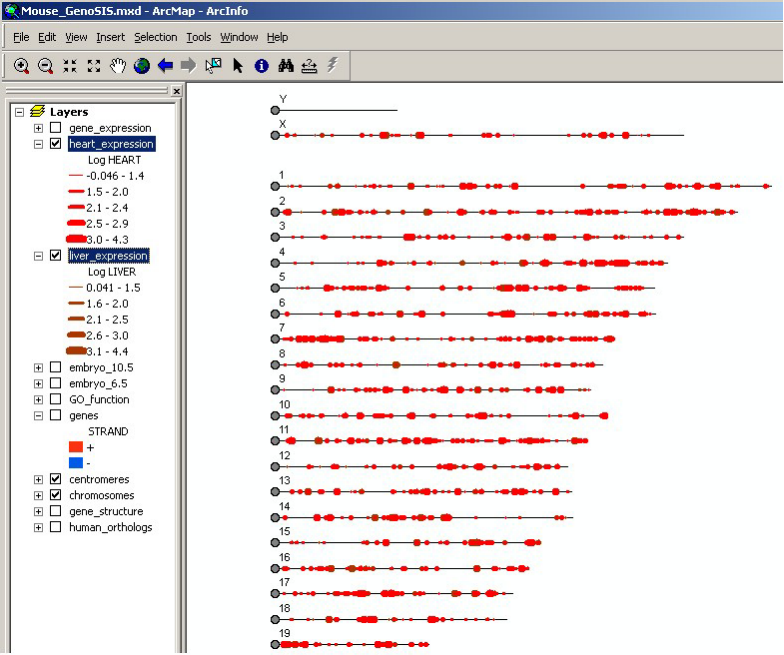
**Visualizing quantitative gene expression data [20] in GenoSIS**. Different colors are used to identify genes associated with each tissue or experiment. The thickness of the symbols is proportional to raw expression levels in the underlying data files.

The representation of the mouse genome using GenoSIS is shown in Figure 2. Each of the genome feature and annotation layers can be selected for display individually or in any combination. The symbols identify the elements in each layer and can be customized and adjusted interactively by the user. Quantitative data, such as gene expression levels, can be visualized using symbols proportional to expression level (Figure 3). One of the strengths of ArcGIS is that it allows the user to create many map layers from a single data source. For example, the *heart_expression *and *liver_expression *layers in Figure 3 were generated from the single *gene_expression *data table. Users have the option of implementing layers with offsets so that the data are displayed as individual tracks instead of direct overlays.

**Figure 4:**
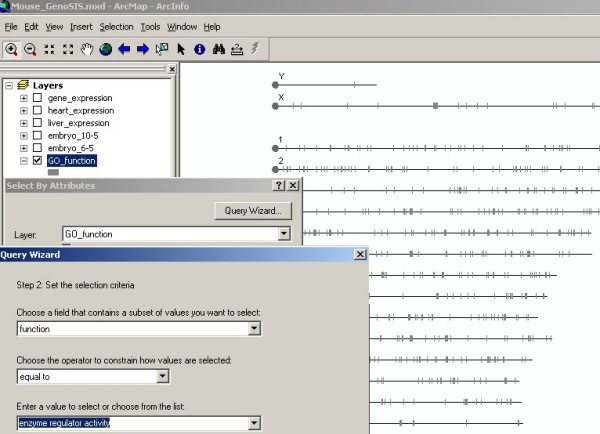
**GenoSIS supports attribute queries**. A simple attribute query can be constructed using the "Select by Attributes" function in the "Selection" menu. For example, "Show a genome wide map of mouse genes that have been annotated to enzyme regulator activity". From the *GO*_*function *layer of 7673 genes with GO molecular function annotation (grey), select genes with enzyme regulator activity function.

**Figure 5:**
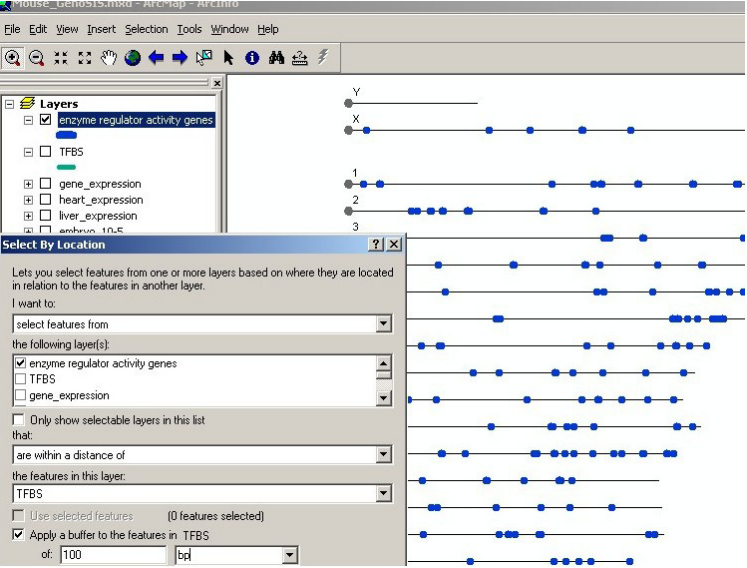
**Query results can be saved as a new fully functional map layer**. The query in figure 4 returns a map of 163 genes (blue), which can be saved as a new map layer "enzyme regulator activity genes." This layer can then be used for other queries including spatial queries. A spatial query can be constructed using the "Select by Location" function in the "Selection" menu. The query wizard allows a user to specify the layers and the spatial relation between them (such as overlaps, contains, within distance of). In this case, the user selects the "enzyme regulator activity genes" and "TFBS" layers, the spatial relation "within distance of" and specifies a "buffer" of the desired distance 100 bp.

**Figure 6:**
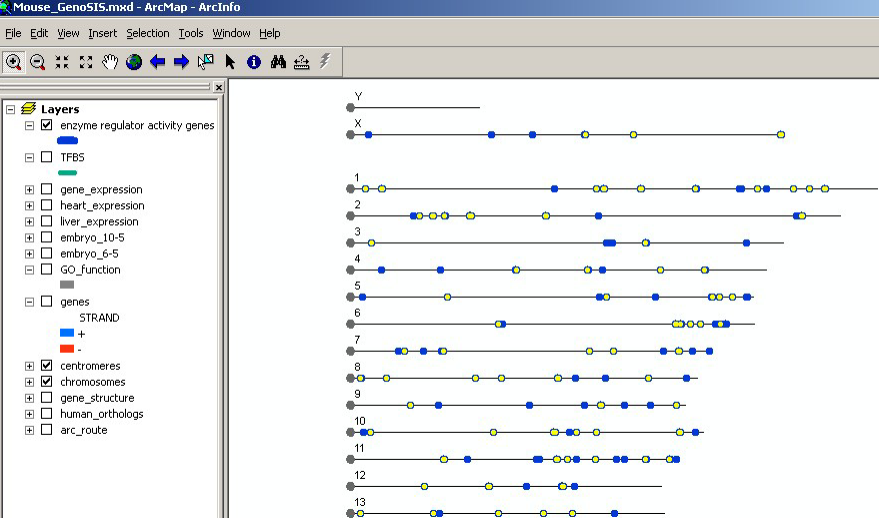
**GenoSIS supports complex queries**. Features in yellow are the result of the query: "Show a genome wide map of mouse genes that have been annotated to enzyme regulator activity and that are located within 100 bp of transcription factor binding sites". Ninety-one of the "enzyme regulator activity genes" meet the spatial query criterion and are shown in yellow. The genome wide view allows a user to see whether or not the selected features appear to be clustered or uniformly distributed across all chromosome regions.

Another feature of GenoSIS is that it supports more than simple keyword queries. Complex queries for the data stored in different layers can be constructed using the "Select by Attributes" and "Select by Location" functions in the "Selection" menu. Figures 4, 5 and 6 demonstrate a complex query based on both data attributes and spatial location: "Show a genome wide map of mouse genes that have been annotated to the GO term, enzyme regulator activity and that are located within 100 bp of transcription factor binding sites." The query is presented as several steps. In figure 4, the *GO_function *layer is made active and a query based on attributes is executed to select genes with enzyme regulator activity function. Figure 5 shows the results displayed as a map, which can be saved and used to create another map layer, "enzyme regulator activity genes." This new layer can then be displayed, queried and manipulated like any other layer. A transcription factor binding sites layer, TFBS, is introduced and used in a spatial query with the enzyme regulator activity genes (displayed in blue). Figure 6 shows the selection of all enzyme regulator activity genes located within 100 bp of transcription factor binding sites displayed in yellow. The genome wide view allows a user to assess whether or not these selected features appear to be clustered or uniformly distributed across all chromosome regions.

## Conclusion

Our primary motivation for developing GenoSIS is to support the use of sequence feature maps for pattern discovery in addition to graphical abstraction of genome content. Our implementation strategy can be used to integrate, visualize, and analyze any data that can be localized on a genome. GenoSIS is unique relative to other genome browsers because of its support for and tight linkage of complex queries and the interactive maps that are the results of such queries. By integrating pattern detection and pattern matching methods directly with genome visualization, GenoSIS can be used as a tool for generating hypotheses about the biological significance of genome feature organization.

## Availability and requirements

• **Project name: **GenoSIS (Genome Spatial Information System)

• **Project home page: **

• **Operating system(s): **Free download of ArcReader for Windows. ArcReader for Linux, Solaris available for a nominal fee.

**Requirements: **Our initial development uses proprietary software, ArcGIS from ESRI. Map files that are published from ArcGIS can be read on Windows platforms by a freely available software tool, ArcReader [11]. ArcReader is also available from ESRI (Environmental Systems Research Institute [12]) for Linux and Solaris platforms for a nominal fee. Relative to ArcGIS, ArcReader provides limited functionality for viewing and querying. We are exploring OpenSource software with full GIS functionality [22,23] that would permit us to distribute software with all of the functionality described in this manuscript without reliance on proprietary software.

## Authors' contributions

The concept for GenoSIS arose from conversations between CJB and MKB when CJB was a Visiting Scholar at the University of Maine's National Center for Geographic Information and Analysis in 1996. MED and CCH jointly implemented the version of GenoSIS described in this manuscript.
